# No evidence so far for the dissemination of carbapenemase-producing *Enterobactericeae* in the community in Switzerland

**DOI:** 10.1186/2047-2994-2-23

**Published:** 2013-09-04

**Authors:** Magdalena Nüesch-Inderbinen, Katrin Zurfluh, Herbert Hächler, Roger Stephan

**Affiliations:** 1 National Centre for Enteropathogenic Bacteria and Listeria, Institute for Food Safety and Hygiene, Vetsuisse Faculty University of Zurich, Winterthurerstrasse 272, 8057 Zurich, Switzerland

**Keywords:** Carbapenemase, Dissemination, *Enterobacteriaceae*, Healthy carriers

## Abstract

**Background:**

Carbapenemase-producing *Enterobacteriaceae* represent an increasing threat to public health and to the treatment of serious nosocomial infections. The aim of this study was to screen for the presence of carbapenemase-producing *Enterobacteriaceae* in human carriers in community settings in Switzerland, a country representative of central Europe.

**Findings:**

Three hundred and fourteen stool samples of healthy staff members of a meat-processing company and 291 fecal swabs from primary care patients were recovered in Switzerland between April 2012 and July 2012 and were tested for carbapenemase-producing *Enterobacteriaceae* isolates by selecting for growth on a carbapenem-containing selective medium. Six resulting isolates *(5 Escherichia coli and 1 Citrobacter youngae)* were subjected to antimicrobial susceptibility tests and PCR analysis by screening for the carbapenemase genes *bla*_OXA-48_, *bla*_VIM_, *bla*_NDM-1_, and *bla*_KPC_ as well as for the extended-spectrum ß-lactamase genes *bla*_TEM,_*bla*_SHV_, *bla*_CTX-M_ and *bla*_CMY-2_. No carbapenemase genes were detected. Resistance to ß-lactam antibiotics was due to carriage of the extended-spectrum ß-lactamase CTX-M-15 in 4 isolates, to CTX-M-14 in one further isolate and to the plasmidic AmpC-ß-lactamase CMY-2 in one isolate.

**Conclusions:**

These results show that carbapenemase-producing *Enterobacteriaceae* are as yet not present in the community. Continuous surveillance is necessary to anticipate future trends in the prevalence and dissemination of carbapenem resistant isolates in the population.

## Findings

The emergence and worldwide spread of carbapenemase producing *Enterobacteriaceae* is of great concern to public health services and a major threat to the efficacy of carbapenem antibiotics such as imipenem, ertapenem or meropenem, which are drugs of choice for the treatment of infections due to extended-spectrum ß-lactamase (ESBL)-producing strains [1].

Currently globally disseminating carbapenem hydrolysing ß-lactamases include the Ambler Class A carbapenemase KPC, the Ambler class B metallo-ß-lactamases (MBLs) such as the IMP-, the VIM- or the NDM-type carbapenemases, and the Ambler class D expanded-spectrum oxacillinases (OXA-type enzymes) [2,3]. The epidemiology of carbapenemase-producing *Enterobacteriaceae*, (especially *Klebsiella pneumoniae*) in European countries follows a pattern typical for hospital-acquired pathogens, and the spread from hospital to community settings is hence a matter of time, as demonstrated in the past by the dissemination of ESBL-producers [4]. Due to the variability of resistance levels to carbapenem antibiotics, carbapenemase-producers cannot be easily detected. Thus, the actual prevalence in the community remains unknown and possibly even underestimated [3].

The aim of the present study was therefore to screen for the presence of carbapenemase-producing *Enterobacteriaceae* in human carriers in a non-hospital setting in Switzerland.

Switzerland, a country with a restrictive antibiotic policy [5], lies not only at a geographical center, but also represents a multicultural socioeconomic and epidemiological intersection in the center of Europe, where differences between linguistic regions reflect the differences observed between surrounding European countries [6]. This makes the country ideal for monitoring temporal-spacial trends in antibiotic resistance in central Europe.

KPC-carbapenemases in *K. pneumoniae* was first reported in a hospital in Switzerland in 2010 [7] attributed to travel-related importation from Italy, followed by four further cases introduced to Switzerland, from hospitalized patients initially treated in Italy and Greece [8]. NDM producing isolates were first reported in Geneva in 2011 [9] and related to travel importation from the Balkans and the Indian subcontinent. In 2012, Oxa-48 producers were identified in Switzerland [10].

Our aim was to determine whether or not, carbapenemase producers have spread beyond the hospital setting.

In an ongoing study of routine stool samples from healthy factory staff and a study on ESBL-producing *Enterobacteriaceae* in fecal swabs of primary care patients [11], 314 stool samples and 291 fecal swabs were obtained and incubated for 24 hours at 37°C in 10 ml of Enterobacteriaceae Enrichment (EE) broth (BD, Franklin Lakes, USA) to enhance the recovery of *Enterobacteriaceae*. One loopful each of the enrichment cultures was inoculated onto chromogenic Brilliance CRE agar (Oxoid, Hampshire, UK) containing a carbapenem to select for carbapenemase producers. Pink colonies (*E. coli*) and blue colonies (*Klebsiella, Enterobacter, Serratia and Citrobacter spp*.) were selected for further analysis. Otherwise pigmented or white colonies were discarded. In total, 6 isolates were collected. Three *E coli* isolates from the 314 stool samples, one *C. youngae* and 2 *E. coli* isolates from the 291 fecal swab samples were identified using the API ID 32 E test (bioMérieux, Marcy l′Etoile, France) and screened by PCR for the presence of *bla*_OXA-48_, *bla*_VIM_, *bla*_NDM-1_, and *bla*_KPC_, using primers described previously [12,13] and using DNA isolated from strains IMMZH201261080, IMMZH201165843, IMMZH201163819 and IMMZH63372-3 as positive controls, respectively (kindly provided by Dr. Guido Bloemberg, Institute of Medical Microbiology, Zürich). The minimal inhibitory concentration of imipenem for the six isolates was performed using Etest IP strips (bioMérieux, Marcy l′Etoile, France), according to the Clinical and Laboratory Standards Institute [14].

No carbapenemase genes were detected in the analysed isolates. Resistance levels to imipenem remained for all isolates below the susceptibility breakpoint of the new interpretive criteria implemented by the CLSI [14], minimal inhibitory concentrations ranging between 0.125 μg/ml and 0.25 μg/ml. Further susceptibility testing performed by disk diffusion assay, using antibiotic disks (Becton Dickinson and Company, Maryland, USA) revealed an ESBL phenotype for 5 of the 6 isolates and an AmpC phenotype for one isolate (data not shown). PCR using appropriate primers [15-17] revealed the presence of the extended-spectrum ß-lactamase CTX-M-15 in 4 isolates, CTX-M-14 in one, and the plasmidic AmpC-type ß-lactamase CMY-2 in a further isolate. Taking into consideration the possibility of reduced carbapenem susceptibility caused by alterations of outer membrane proteins in combination with an ESBL- or an AmpC-type enzyme as decribed previously [18,19], these results offer an explanation for the initial growth of these isolates on the selective medium.

In conclusion, our study testifies the current absence of carbapenemase-producers in healthy people and primary care patients in Switzerland, indicating that carbapenemase-producers have not entered the community in this country. Additional studies should be carried out in future in order to continuously evaluate the dissemination of carbapenemase-producers among enteric bacteria in the population, including screening of patients transferred from hospitals from countries where carbapenemase-producers have been detected. Utmost care should be taken to minimize the impact of the emerging crisis of carbapenem resistant *Enterobacteriaceae*.

## Ethical approval

The sampling of primary care patients was approved of by the local ethics committee of Zurich and is registered as number KEK-StV-Nr. 54/12.

## Consent

Informed consent was obtained from the patient for the publication of this report and any accompanying images.

## Competing interests

The authors declare that they have no competing interests.

## Authors’ contributions

RS, HH and MNI conceived the study and MNI drafted the manuscript. KZ participated in the design of the study and carried out the microbiological and molecular biological tests. All authors read and approved the final manuscript.
